# Ultra-Resolution Spectral Correction Based on Adaptive Linear Neuron for Biomedical Signal Processing

**DOI:** 10.3389/fpubh.2021.682377

**Published:** 2021-05-14

**Authors:** Binqiang Chen, Baixun Zheng, Weifang Sun

**Affiliations:** ^1^School of Aerospace Engineering, Xiamen University, Xiamen, China; ^2^College of Mechanical and Electrical Engineering, Wenzhou University, Wenzhou, China

**Keywords:** fast fourier transform, biomedical signal, adaptive linear neuron, sinusoidal wave, spectrum correction

## Introduction

Sinusoidal waves (SWs) are often presented in engineering dynamic measurements. They also appear in biomedical signals (1–3), such as electrocardiogram (ECG), electroencephalogram (EEG) and electromyography (EMG). The power line interference (4–6) is among the major types of interferences in these biomedical signals and it should be removed using hardware or software based techniques (7–9). A simple harmonic wave can be characterized using a group of harmonic parameters. By identifying these parameters, a compensation signal can be constructed and subtracted from the original measurement (10). In the literature, current techniques to estimate harmonic parameters mainly rely on discrete Fourier analysis. According to the theory of discrete Fourier analysis, the spectral resolution of a spectrum is the reciprocal of the signal sample length. Therefore, greater values of the sampling length are beneficial in improving the performance of harmonic parameter identification.

During the past decades, various spectrum correction methods have been developed to estimate harmonic information with high precision (11–15). With the help of spectrum correction methods, the harmonic information of a signal SW can be retrieved effectively. In the practices of biomedical signal processing, multiple SWs may be presented in the signal simultaneously. In ideal cases, the frequency distances of adjacent SWs should be sufficiently large such that they can be separated individually. Generally, at least a distance of five spectral resolutions is required. Otherwise, as the distance between their frequencies gets closer, the PFE even deteriorates and the overlapping of their energies cannot be neglected. To separate MSWs with relative small differences in frequency, there are still no effective method in the literature.

In order to address this problem, an effective solution is to predict additional data samples beyond the recorded datasets. As the number of samples increases, including the original samples and the predicted samples, the spectral resolution in the FFT spectrum can be improved such that the coupled MSWs can be separated. This solution has been adopted in the theory of modern spectrum analysis (16). However, current techniques can only ensure effective time series prediction in a short time. The prediction errors increases dramatically in a long term situation. As such, only a limited number of prediction samples can be obtained by post-processing of the original dataset. Hence, a limited improvement of spectral resolution can be derived.

In this paper, a novel separation method, based on the adaptive linear neuron, is proposed for MSWs whose frequencies are closed spaced in the spectral domain. The autoregressive model is employed to describe intrinsic structure of the MSW data. The obtained samples are utilized to train the AR model. In order to reduce the training errors, a norm function is formulated and the pseudo inverse is used to derive the closed form solution. Additional samples can be predicted via the established AR model from the recorded samples. Simulation results show the auto-regression algorithm can guarantee precise predictions in a long time interval beyond the original sampling length. An extremely high spectral resolution can be achieved from the extended data length, therefore the coupled MSWs can be distinguished in the spectral domain. The proposed method can be regarded as a theoretical model to separate the coupled MSWs in the frequency domain. Numerical simulations are presented to show the effectiveness of the proposed method.

## Perspective Method for Separation of Closed Spaced MSWS

### Picket Fence Effect and Aliasing of MSW

In mathematics, FFT generates a series of spectral lines evenly spaced at a specific frequency resolution, which is the reciprocal of the entire sampling interval. For a sinusoidal wave with a specific frequency, If the signal sampling period does not contain an integer number of harmonic periods (full period sampling condition), the energy leakage problem of the harmonic components occurs. This phenomenon is also known as the picket fence effect (PFE). Side effects of PFE are also reported in wavelet decompositions (17, 18).

### Adaptive Linear Neuron

**Figure 1:**
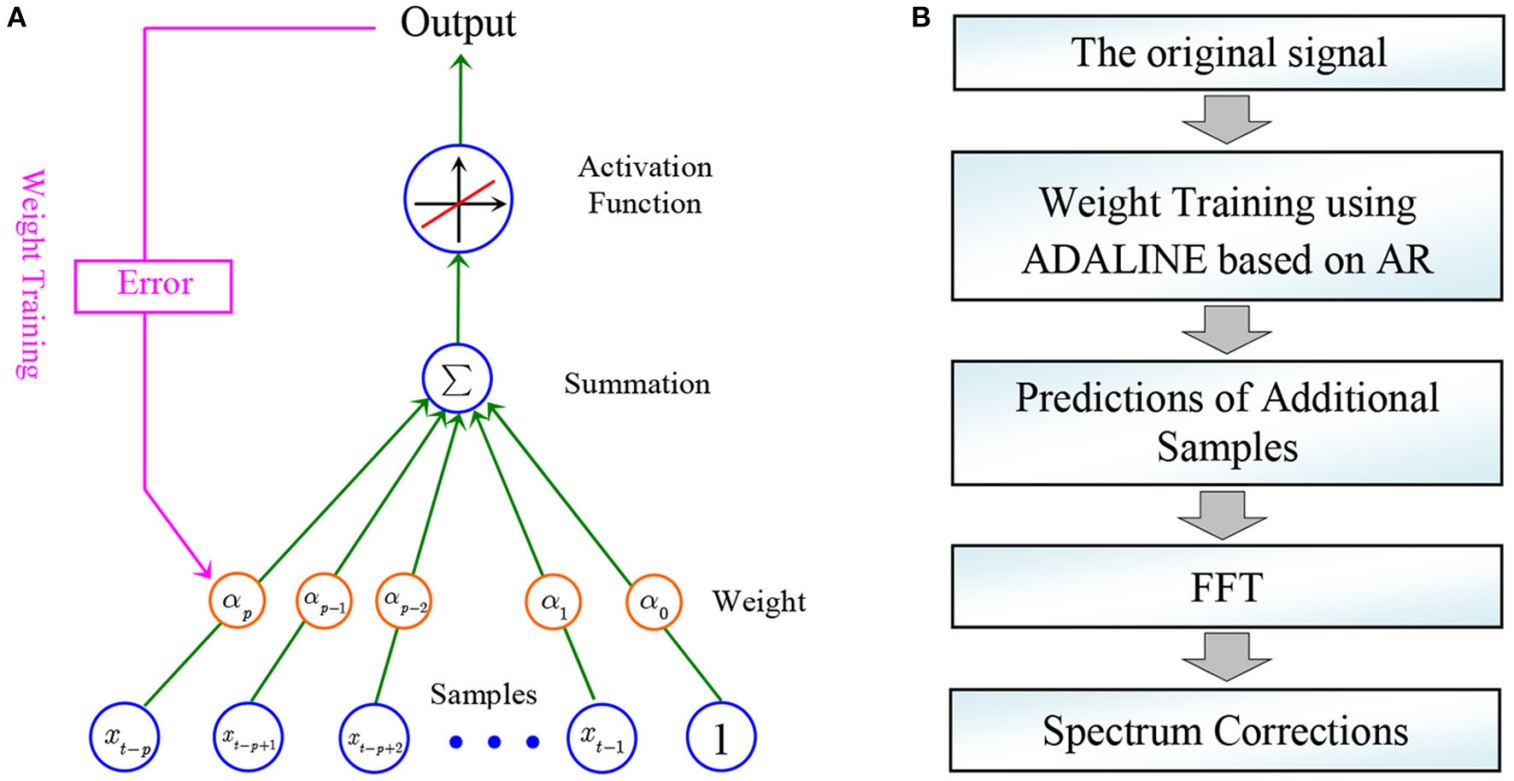
**(A)** The structure of a typical adaptive linear neuron; **(B)** The procedure of the proposed method.

The adaptive linear neuron is a naive and useful tool in artificial neural networks (19, 20). As a single layer artificial neural network, the structure of a typical ADALINE is illustrated in Figure 1A. A classical proportional function *y* = *x* is employed as the activation function. On the other hand, the indicator of mean squared error can be employed as the loss function. As a supervised learning method, the strategy of back propagation (BP) can be used to train the weights. The research of artificial neural network is a hot topic in the scientific and engineering community. In the past decade, by introducing new activation functions and loss functions, deep neural network based on stacked neurons can be constructed.

### A Perspective Method for Multi-SW Separation Problem

In order to establish a precise model for a dynamic process of multi-SW, the celebrated auto regressive model is introduced to implement weight training in ADALINE. An autoregressive model of order *p* can be expressed as

(1) $$\begin{array}{r} x_{t}=\alpha_{0}+\alpha_{1}x_{t-1}+\alpha_{2}x_{t-2}+\ldots +\alpha_{p}x_{t-p}+\varepsilon_{t}. \end{array}$$

The error term ε_*t*_ can be interpreted as stochastic noises. For an MSW series with a mean value of zero, this parameter can be set to zero. The values of rest weight parameters α_*i*_, *i* = 0, 1, …, *p*, can be obtained using a least mean square (LMS) approach. Supposing that we have an original series of length *L*. All the equations associated with the AR(*p*) model in Equation (1) can be expressed in a matrix form as

(2) $$\left(\begin{array}{cccc} x_{1} & x_{2} & \ldots & x_{p}\\ x_{2} & x_{3} & \ldots & x_{p+1}\\ \vdots & \vdots & \ddots & \vdots \\ x_{L-p} & x_{L-p+1} & \ldots & x_{L-1} \end{array}\right)\left(\begin{array}{c} \alpha_{1}\\ \alpha_{2}\\ \vdots \\ \alpha_{p} \end{array}\right)=\left(\begin{array}{c} x_{p+1}\\ x_{p+2}\\ \vdots \\ x_{L} \end{array}\right)$$

In the above equation, the coefficient matrix *A* is of the size (*L*−*p*) × *p*. It is a matrix of full column rank and its pseudo inverse can be computed as *A*^†^ = (*A*^*T*^*A*)−1*A*^*T*^. The unknown column vector containing the AR parameters can be computed using

(3) $$\begin{array}{r} \alpha =A^{†}{\left(\begin{array}{c} x_{p+1}x_{p+2}\ldots x_{L} \end{array}\right)}^{T}. \end{array}$$

The above least mean square method is among the various techniques to estimate the AR parameters. Because the order of *p* is relatively small, this method can be extremely efficient.

After establishing the AR model, data extrapolation can be made. Beside the available data, additional samples can be predicted using Equation (1). The flow chart of the proposed method is displayed in Figure 1B. The estimation error significantly affects the prediction accuracy, which is shown in the discussion part.

Since the length of the time series being investigated can be substantially enlarged using the data extrapolation, the spectral resolution in the FFT spectrum also improves. As a result, the MSWs closely spaced in the original spectrum can be separated in the new spectrum.

### Discussions

In the proposed method, the training process of ADALINE is completed by AR. However, in the literature, the back propagation (BP) is more popular. By using BP, the cost function can also be blow in value. However, the prediction errors grow significantly as the prediction length increases. Therefore, AR can be regarded as an effective long term prediction tool in multi-SW processes.

We can lengthen the entire sampling interval based on predictions of additional samples, which significantly improves the actual frequency resolution in the FFT spectrum. That is to say, we can separate their counterparts in the spectral domain. However, for SW components which do not satisfy the full period sampling condition, spectrum correction methods should be combined to further investigate their harmonic information.

## Author Contributions

BC and WS conceived and designed the classification method, reviewed, and edited the manuscript. BZ performed the experiment, preprocess, analyzed the data, and wrote the manuscript. All authors read and approved the manuscript.

## Conflict of Interest

The authors declare that the research was conducted in the absence of any commercial or financial relationships that could be construed as a potential conflict of interest.
